# Why We Fail

**Published:** 1882-02

**Authors:** 


### HOMŒOPATHIC PHYSICIAN.

A MONTHLY JOURNAL OF MEDICAL SCIENCE.

"If our school ever gives up the strict inductive method of Hahnemann, we are lost, and deserve only to be mentioned as a caricature, in the history of medicine."—CONSTANTINE HERRING.

Vol. II. FEBRUARY, 1882. No. 2.

### EDITORIAL.

WHY WE FAIL. In a recent number of the *New York Medical Times*, we read the following:

"Be it at once admitted that our temptations to laxity in these particular studies [Anatomy, Physiology and Chemistry], are actually inherent in much of Hahnemann's teaching. He entertained a just contempt for the 'Science' of his day, but in ignoring it he kicked aside the very ladder by which he had risen into eminence. He overlooked the prime secret of his own strength—his multifarious knowledge, and in his victories he ignored his adjuvants. He taught that we may know disease only by its symptoms, and his *dictum* spawned 'symptom-coverers' who have essayed to take the places of the 'Old Guard.' We often hear it said to-day that modern homœopaths do not realize such 'cure-work' as did the pioneers. Why is this? We have the same materia medica in even more available shape, and, happily, with many a tare which vexed them uprooted for us, and yet we fall behind them in actual puissance! We are as ambitious as they; as industrious; we have even the stimulus of their example, and yet we are obliged to acknowledge—'there were giants in those days!'

"Is their ability anything other than this—that they crowned the fullness of medical knowledge with the divine secret of the *similars?*"

In pointing out the erroneous impression conveyed in these lines, we have no desire to enter into any controversy with their ingenious (though scarcely always ingenuous) author. His ability to handle any side of any question has often excited our liveliest admiration, and we have felt *proud* to think our school possessed such a *champion.* Indeed, his advocacy of Homœopathy, to quote his own words, only

"gives me indomitable courage, for if Homœopathy were not a staunch ship freighted with a truth which the Omnipotent will not let perish, she never could have carried the barnacles that fatten on her hull."

But let us not give vent to our *pride* over this gentleman; excessive pride is sinful! Rather let us turn to other and important matter. In the above quotation we would remark especially upon the following:

"We often hear it said that modern homœopaths do not realize such 'cure-work' as did the pioneers. Why is this? * * * Is their ability anything other than this, that they crowned the fullness of medical knowledge with the divine secret of the *similars?*"

In the first place, we are somewhat surprised that great learning should be claimed for Hahnemann and his collaborateurs. We are rather more accustomed to hear them classed as ignoramuses, and such like. Indeed, we have even heard it said they knew nothing of medical science, and were only fit to compare symptoms, wherefore they were mockingly called "symptom-coverers," as a term of opprobrium. In view of these facts, it may well be imagined that this new explanation of their successes strikes us as peculiar. The truth is, the successes of these "giants" were so grand, so astonishing, that they could not be hid, still less could they be denied, hence they must be explained away and laid to anything rather than the true source—*symptom-covering.*

The impression desired to be made by the writer of this quotation (at least as we read it), is that modern homœopaths fail to emulate the successes of the "pioneers," because they are not so skilled in medical lore as were their sires in science. This we believe to be the minor reason for lack of "cure-work." We acknowledge that they, who "crown the fullness of medical knowledge with the divine secret of the *similars,*" will be the best of healers—*provided* they use such knowledge properly. This proviso is most important; since, however helpful any branch may be in its proper place, it can only do harm if strained to improper functions. So too we all acknowledge that the homœopathic physician should be thoroughly educated in medicine and its allied sciences; he can never be too well educated; but let him beware how he uses this knowledge, if he desire success.

Such knowledge must be used as the law of the similars directs; not as something extraneous and foreign to that law. Moreover, it must be borne in mind that the theories of physiology, pathology, etc., are mere human interpretations of things seen, and have not the certainty and infallibility of Nature's law. Knowledge is only dangerous when it may be classed as "too little." The present state of knowledge of these branches certainly cannot be called other than "too little." Notwithstanding the uncertainty which attends physiological and pathological knowledge, at present, it cannot be fairly claimed that modern homœopathists fail *only* because they are less learned in anatomy, chemistry and physiology than were their predecessors. Says our writer: "He (Hahnemann), entertained a just contempt for the 'Science' of his day, but in ignoring it, he kicked aside the very ladder by which he had risen into eminence." One might well ask, how could a science which was justly deserving of contempt help any one to perform wonderful cure-work? So by his very words, we see it was not science, as *he* terms science, which enabled these men to do such grand cure-work, and to revolutionize a medical world.

The pioneer homœopaths—Hahnemann, Bœnninghausen, etc.—were successful because they practiced Homœopathy; we of to-day fail because we do *not* practice Homœopathy. The pioneer homœopaths were successful because they were thorough symptom-coverers; we of to-day fail because we are *not* thorough symptom-coverers. Let any one who will, compare a case reported by a "pioneer" with one reported by a modern homœopath; the cause of the wonderful success of the one, and the reason for the stupid failure of the other will then be apparent to the most obtuse. "Fullness of medical knowledge" coupled with a rare sagacity taught these early pioneers of Homœopathy, to discriminate between law and guess, between fact and theory, between the useful and the useless. Hence these men, who our friend fitly acknowledges "were giants in those days," became *symptom-coverers.* And in later days, a Hering and a Dunham, though crowned with "the fullness of medical knowledge," were proud to be classed among the *symptom-coverers.*

Said Dr. Dunham:

"Those of our School who insist upon pathology as a *basis* of therapeutics, who look upon the single objective symptom and its nearest organic origin as the subject for treatment, and who deride the notion of prescribing upon the totality of the symptoms, and claim to be more than mere symptom-coverers, in that they discover and aim to remove the *cause* of the disease—these colleagues are as false in their pathology, according to the highest old school authorities, as they are faithless to the doctrines, and impotent as to the successes, of the founder of the homœopathic school."*

Dr. Dunham here tells us why we fail to "realize such 'cure work' as did the pioneers." Let us repeat his words: "Those * * * who claim to be more than mere symptom-coverers, in that they discover and aim to remove the *cause* of the disease—these colleagues are as false in their pathology, * * * as they are faithless to the doctrines, and impotent as to the successes of the founder of the homœopathic school.'' Failure to cure is not charged, by Dr. Dunham, to ignorance of chemistry or anatomy or physiology, but he declares that those "who deride the notion of prescribing upon the totality of the symptoms, and claim to be more than mere symptom-coverers" are "impotent as to the successes of the founder [and of course, also of those of his colleagues of the 'Old-Guard'] of the homœopathic school." Did not Dr. Dunham know the proper value of these branches of medicine? Was he a symptom-coverer from ignorance or from conviction? Were the symptom-coverers Hering and Dunham failures as to healing the sick or were they successful? If successful, did they claim their successes were due to their knowledge of chemistry, physiology, pathology, etc., or to their symptom-covering style of prescribing? The answers to these questions are self-evident, to all who knew these gentlemen.

While "fullness of medical knowledge" led such men as Hering and Dunham to acknowledge symptom-covering as the only true method of homœopathic prescribing, a lack of such medical learning does not prevent one from attaining great success in healing. Homœopathy presents a notable instance of this in the person of the late Dr. Von Bœnninghausen, "the peerless prescriber of Munster," whose practical successes have never been excelled. The secret of Bœnninghausen's success was his laborious and minute examination of his cases and his thorough knowledge of the materia medica. To illustrate this clearly, we quote the following case from the preface to his "Therapeutic Pocket-Book":

"E. N., of L., a gentleman about 50 years of age, of a healthy, almost too, florid complexion, generally of a cheerful temper, during severer attacks, however, inclined to break out into fits of passion, with a decided nervous ir-

* See *Homœopathy, The Science of Therapeutics*, p. 114.

ritability, having been allopathically cured of a rheumatic pain, as it was called, in the right eyehole by external remedies, which, however, could not be ascertained, suffered for the last months from a peculiar kind of violent pain on the lower part of the right thigh, comprising all the muscles of the back part of it, viz. from the calf down to the heel, with the exception of the joints of the knee and ankle. The pain itself he describes as a very acute, crampy, jerking, tearing one, often interrupted by stitches, proceeding from the inner part: in the morning, however, the pain upon the whole is much less, but rooting, and the patient feels as if beaten all over. It increases towards evening and in a quiet state—particularly after motion, in sitting and standing, and particularly doing so during a walk in the open air. During the walk itself the pain often flies from the right calf to the upper part of the left arm and is again most insupportable, if the patient puts his hand into his pocket or bosom and keeps the arm quiet: by moving the arm the pain is lessened and often quickly returns to the right calf. He finds the greatest relief in moving up and down in the room and rubbing the affected part. The concomitant complaints consist in sleeplessness before midnight, often repeated attacks of a quickly over-running heat with thirst in the evening, without a preceding shivering, a disagreeable, greasy taste in the mouth, with nausea in the throat and an almost continual, pressing pain in the lower part of the chest and the pit of the heart, as if something were forcing its way through.

"No homœopathic physician, who is familiar with the effects of his remedies, having so complete and so accurate a picture of the disease before him, will long be in doubt as to the most suitable remedy in this case, as all those signs together answer only to one of them homœopathically; the beginner on the contrary will be obliged to look for almost every single symptom and will only after a long investigation find the right one amongst the concurring medicines. Between these two extremes of knowing and not knowing there are numerous degrees of half-knowledge, which require a more or less frequent consultation of the Manual.

"One, for instance, knows, that those pains, which fly from one part to another, being worse towards evening and in repose, the greasy taste in the mouth, sleeplessness before midnight and some other of the symptoms, which have been mentioned, are pre-eminently the effect of *Pulsatilla:* he is, however, not sure, whether also the rest of the symptoms belong to it, and wishing to act conscientiously, he will not mind the trouble of comparing the same, when he will soon find that *Pulsatilla* cannot be the right homœopathic remedy, as many of the symptoms, besides those of the mind, are not similar to *Pulsatilla*, nay even are directly opposed to it.

"Another has studied more the peculiarities of pains and clearly recollects that the principal effects of *China* are laming, crampy, tearing pains, as if from being beaten, stitches, coming from interior towards exterior parts, as well as pains, flying from one part to another: he believes, moreover, that other symptoms, as sleeplessness before midnight, aggravation in quiet amelioration through moving and rubbing, flying heat with thirst, are also caused by *China:* but with regard to the other symptoms he is not sure and he is therefore obliged to consult a repertory, when he will soon, like the first, meet with contradictions, proving to him, that in the present case *China* is not the fit remedy.

"Now, neither of them will think of making a trial and of giving the patient a remedy, the effect of which, as in this case, is so uncertain; but as conscientious homœopathic physicians they will consider and compare (and with the assistance of this Pocket-Book they will soon find), without a great deal of trouble, the only fit and truly homœopathic remedy.

"But suppose, there was a third, who understood enough of Homœopathy, to know at once that *Pulsatilla, China* and other concurring remedies are not the right medicines, but who does not sufficiently know *Valeriana,* which corresponds to the principal symptoms, and therefore is doubtful as to the fitness of this less often used remedy: he will only have to look for some dubious symptoms to convince himself that this must be the best amongst all known medicines, which was proved by the result; as after a single, highly diluted, very small dose, dissolved in water, the principal complaint with all the concomitant ones was within three days completely removed."

To further illustrate the minuteness of Bœnninghausen's analysis of symptoms, let us note the many divisions he makes of influence of motion upon patients' symptoms. Under various headings, given in his "Therapeutic Pocket-Book," there are no less than fifty-five different conditions of motion, etc., from which aggravations ensue. This, it will be remembered, is only one modality; others are analyzed equally as thoroughly. Contrast this careful and thorough manner of analyzing symptoms with the careless and hasty methods in vogue at present, and one will no longer wonder that we "do not realize such 'cure-work' as did the pioneers." Were we of to-day to collect the symptoms of our patients as thoroughly, to analyze them as carefully, to make the materia medica our chief study, as did Bœnninghausen and others of the "pioneers," we would soon realize their wonderful "cure-work." Symptom-covering was their simple but laborious method. Being both simple and laborious, it has not been extensively copied.

